# Diagnostic accuracy of multi-organ point-of-care ultrasound for pulmonary embolism in critically ill patients: a systematic review and meta-analysis

**DOI:** 10.1186/s13054-025-05359-x

**Published:** 2025-04-23

**Authors:** Rafael Hortêncio Melo, Luciana Gioli-Pereira, Igor Dovorake Lourenço, Rogério Da Hora Passos, Adriana Tumba Bernardo, Giovanni Volpicelli

**Affiliations:** 1 Department of Critical Care, Hospital Municipal Vila Santa Catarina Dr.Gilson de Cássia Marques de Carvalho; Hospital Israelita Albert Einstein, São Paulo, SP Brazil; 2 Department of Critical Care, Hospital Israelita Albert Einstein, São Paulo, SP Brazil; 3 Da Vita Kidney Treatment, São Paulo, SP Brazil; 4 Complexo Hospitalar de Doenças Cardiopulmonares Cardeal Dom Alexandre Do Nascimento, 47QM+FCJ, Av. Pedro de Castro Van-Dúnem Loy, Luanda, Angola; 5 Department of Medical and Surgical Science, Magna Graecia University, Catanzaro, Italy

**Keywords:** Point-of-care ultrasound, Pulmonary embolism, Diagnostic accuracy

## Abstract

**Background:**

The clinical presentation of acute pulmonary embolism (PE) can range from mild symptoms to severe shock, circulatory arrest and even death, thereby presenting with a significant high mortality when undiagnosed. Computed tomography pulmonary angiography (CTPA) is the gold-standard imaging modality for diagnosing PE, however, it has several practical limitations and is not widely available in low-income country settings. In this context, point-of-care ultrasound (POCUS) has emerged as a valuable bedside, non-invasive diagnostic tool. This meta-analysis assesses the accuracy of multi-organ POCUS for diagnosing PE in critical care settings.

**Study design and methods:**

We conducted a systematic search of Pubmed, Embase, Scopus and the Cochrane Library databases for studies comparing multi-organ POCUS with CTPA or ventilation-perfusion scans for PE diagnosis in critical care departments. Two reviewers independently completed search, data abstraction and conducted quality assessment with QUADAS-2 tool. Heterogeneity was examined with I^2^ statistics. We used a bivariate model of random effects to summarize pooled diagnostic odds ratio (DOR), sensitivity, specificity, positive likelihood ratio (PLR), negative likelihood ratio (NLR) and summary receiver operating characteristic (SROC).

**Results:**

Four studies met the inclusion criteria, comprising 594 patients. The mean age of participants ranged from 55.2 to 71 years. Prevalence of PE ranged from 28 to 66.2%. CTPA was the primary reference standard used in most studies. Multi-organ POCUS for PE diagnosis demonstrated a pooled DOR of 25.3 (95% CI 4.43–82.9) with a pooled sensitivity of 0.90 (95% CI 0.85–0.94; I^2^ = 0%) and specificity of 0.69 (95% CI 0.42–0.87; I^2^ = 95%). The PLR was 3.35 (95% CI 1.43–8.02) and the NLR was 0.16 (95% CI 0.08–0.32). The SROC curve showed an AUC of 0.89 (95% CI 0.81–0.94).

**Conclusions:**

Multi-organ POCUS has high diagnostic accuracy for PE diagnosis in critically ill patients. Further research is needed to validated these findings across different patient populations.

**PROSPERO registration:**

CRD42024614328.

**Graphical abstract:**

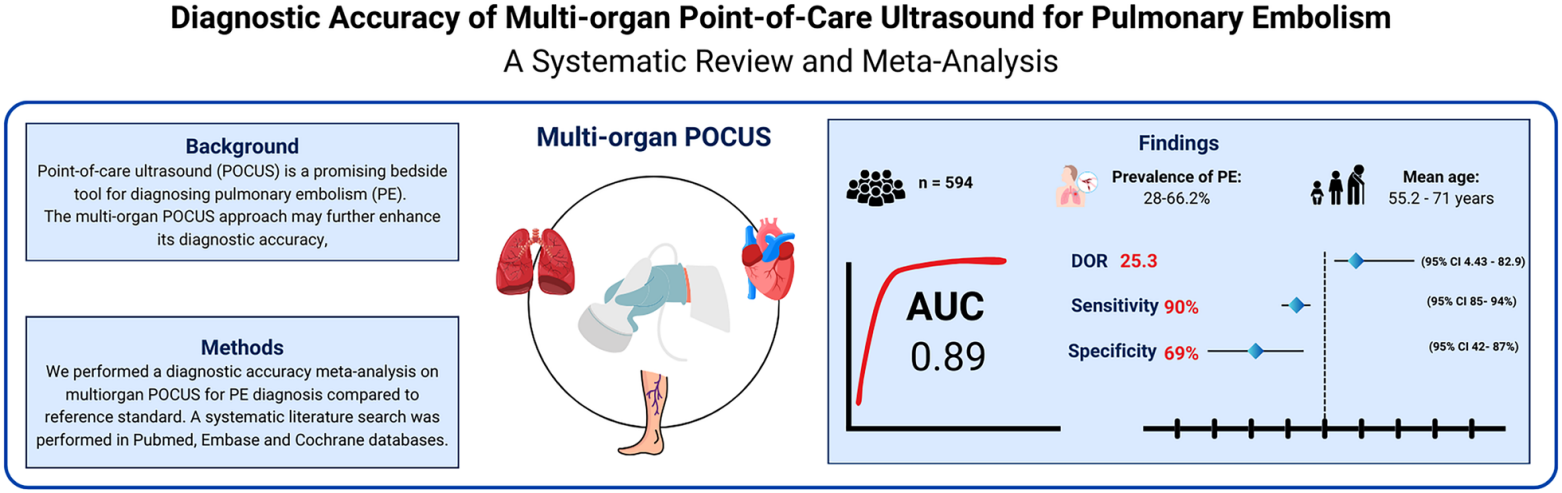

**Supplementary Information:**

The online version contains supplementary material available at 10.1186/s13054-025-05359-x.

## Background

The clinical presentation of acute pulmonary embolism (PE) can range from mild symptoms to severe shock, cardiac arrest and even death [1–3]. Common symptoms include sudden onset dyspnea, chest pain, syncope, and hemoptysis, with dyspnea being the most frequently reported symptom, occurring in a significant majority of patients [2]. This heterogeneity in presentation can be attributed to several factors, such as underlying cause of PE, location and load of thrombus, number of pulmonary lobes affected and the presence of comorbidities [4, 5]. These factos makes the diagnosis of PE challenging, requiring an accurate diagnostic process with a high level of clinical suspicion and a structured stepwise approach. Owing to the nonspecific nature of symptoms and signs, clinical prediction rules such as the Wells score, revised Geneva score, and Pulmonary Embolism Severity Index (PESI) can assist in risk stratification and guide decisions regarding further diagnostic testing [1].

Computed tomographic pulmonary angiography (CTPA) is considered the gold standard for PE diagnosis because of its high sensitivity and specificity [6]. However, the CTPA has several practical limitations in austere scenarios, such as with hemodynamically unstable patients and in limited-resource settings, which can affect its utility. These include high cost, the logistical challenges of transporting an unstable patient to the radiology department, risk of radiation exposure in pregnant patients and limited availability, particularly in low-income countries.Additionally, the use of iodinated contrast material increases the risk of nephrotoxicity and allergic reactions, especially in patients with pre-existing renal impairment or contrast allergies [4].

When CTPA is not feasible, point-of-care ultrasound (POCUS) has demonstrated its usefulness in clinical practice for ruling in or out PE. Each modality—lung ultrasound, leg vein ultrasonography and focused echocardiography—has been independently shown to be useful and accurate method for confirming the diagnosis of PE [7–10]. However, despite their utility in specific clinical settings, POCUS of each isolated organ system has relatively low sensitivity. As a result, none of these methods alone can reliably rule-out PE. The application of multi-organ POCUS, which combines lung, cardiac and venous ultrasound, has demonstrated increased diagnostic sensitivity for PE in some studies compared with single-organ approaches [11]. A recent meta-analysis [12] evaluated the accuracy of each organ ultrasound in diagnosing PE, but did not assess the performance of multi-organ POCUS approach. Therefore, we conducted a systematic review and meta-analysis on the accuracy of multi-organ POCUS for diagnosing PE in critical care setting.

## Methods

This systematic review and meta-analysis was performed and reported in accordance with the Cochrane Collaboration Handbook for Systematic Review of Interventions and the Preferred Reporting Items for Systematic Reviews and Meta-Analysis (PRISMA) Statement guidelines [13, 14]. This meta-analysis involved secondary data from published studies, exempting it from institutional review board approval.

### Inclusion criteria

We selected articles assessing the accuracy of combined lung, cardiac and venous (multi-organ) POCUS for the diagnosis of PE. The study population included patients of age ≥ 18 years with suspected PE. Two reference standards for the diagnosis of PE were accepted: CTPA and ventilation/perfusion (V/Q) scan. Moreover, the included studies were required to have a 2 × 2 table of true positive, false negative, true negative, and false positive counts, either extracted from the original article or calculated from other reported information. We excluded preclinical studies, studies including pediatric populations, case reports, conference abstracts, opinion articles, editorials and non-English articles.

### Search strategy

A systematic literature search was performed in the following databases: PubMed, MEDLINE/Embase, Scopus and the Cochrane Library. The search strategy included combined terms such as ‘’pulmonary embolism’’, ‘’ultrasound'’ and CTPA/ventilation-perfusion scan-related terms. The detailed string is available in the Supplementary Material. Additionally, a backward search (snowballing) and a forward search (citation-tracking) were conducted for the included articles and relevant literature review. If the required data were not available in the published studies, we contacted the corresponding author to obtain the information.

### Study screening and selection

Two authors (R.M. and L.G.) independently screened titles and abstracts and then screened the full texts of the selected articles to identify eligible studies. Any disagreements were resolved through discussion with a third author (R.P). Rayyan.ia [15] software was used to screen, select and exclude duplicate studies.

### Data extraction

Each included study was independently scrutinized by two authors (R.M. and L.G.) to obtain the following data: study design, sample size, ultrasonography technique, year, country, median population age, sex proportion, prevalence of PE, diagnosis, reference standard for PE diagnosis, and sensitivity and specificity of multiorgan POCUS for the diagnosis of PE.

### Risk of bias assessment

Two authors (R.M. and I.D.) independently performed the Quality Assessment of Diagnostic Accuracy Studies-2 tool (QUADAS-2) [16] to evaluate the risk of bias,which was tailored to suit the review question. Signaling questions were used to assess the following domains: patient selection, index test, reference standard and flow and timing. The risk of bias was assessed across each of the 4 domains and applicability across the first 3 domains. If a study had at least one high-risk domain or two moderate-risk domains, it was rated as having an overall high risk of bias. Disagreements about quality assessment were resolved by consensus by an additional author (L.G.).

### Statistical analysis

We performed a meta-analysis of the studies using the reference standard of each study for PE diagnosis. Diagnostic effect measures were obtained from 2 × 2 contingency tables to calculate the sensitivity, specificity, diagnostic odds ratio (DOR), positive likelihood ratio (PLR) and negative likelihood ratio (NLR) with 95% confidence intervals (95% CI). To account for heterogeneity in methodology and demographics across studies, a bivariate random-effects model was used, and forest plots were generated for graphical representation. We constructed summary receiver operating characteristic (SROC) models and calculated the area under the curve (AUC).

We quantified the heterogeneity of the included studies using the inconsistency index (I^2^). Publication bias was assessed by analyzing funnel plot asymmetry and performing Egger’s test. Statistical significance was assumed for *p* < 0.05. Statistical analysis was carried out via R software/environment (version 4.4.0, R foundation for Statistical Computing).

## Results

### Study characteristics

**Fig. 1:**
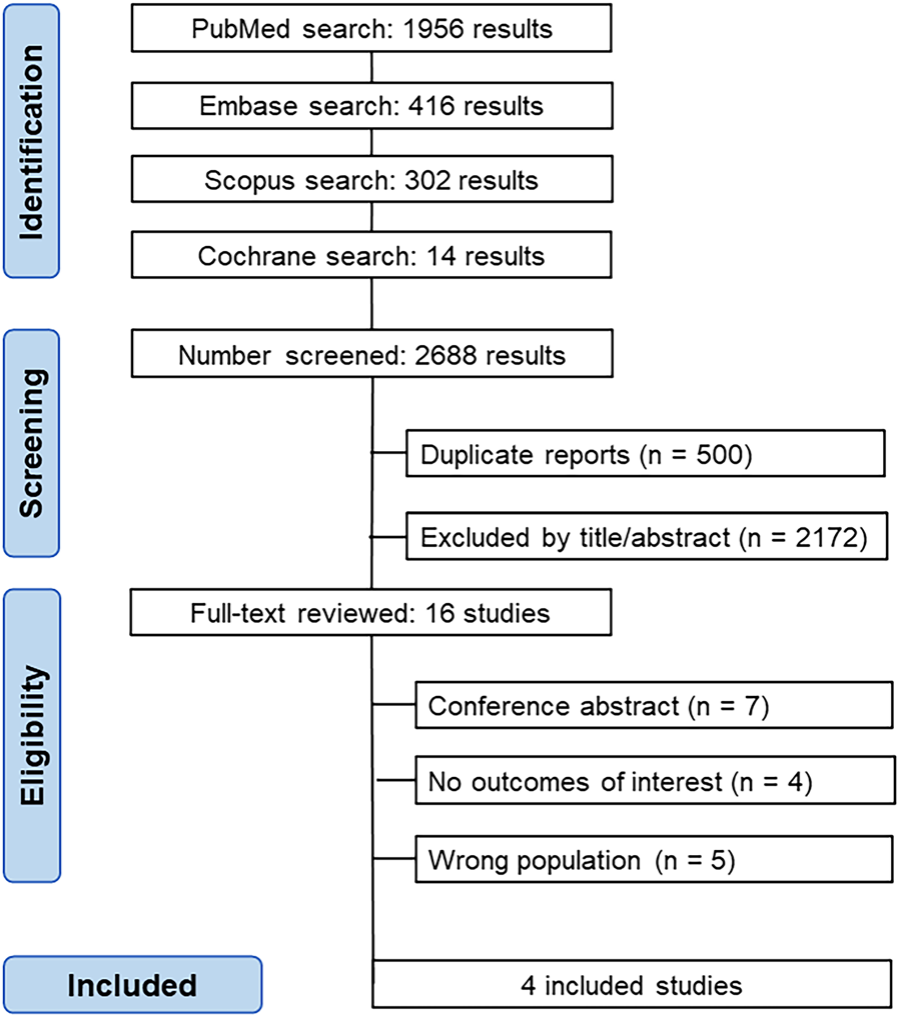
PRISMA flow diagram of study screening and selection

As shown in Fig. 1, the initial search yielded 2688 results. After removing duplicate records and excluding ineligible studies, 16 studies remained for full-text review on the basis of the inclusion criteria. Following further examination, 4 studies were ultimately included, encompassing a total of 594 patients.

The characteristics of the included studies are summarized in Table 1. All studies employed a prospective design. The mean age of participants ranged from 55.2 to 71 years, with the female prevalence varying from 20 to 55%. The prevalence of PE among the study populations ranged from 28 to 66.2%.

**Table 1:** Baseline characteristics of included studies

Study	Design	Location	Population	Sample size	Female, n (%)	Age, years (mean)	PE prevalence (%)	Wells score (mean)	Mean D-dimer (mcg/dl)	Sonographer	Reference Standard
Aktürk 2017 [17]	Prospective	Netherlands	ED	92	40 (43.2)	55.2 ± 17.4	66.2	4.9	3.6	Experienced chest physician with specific training	CTPA
Falster et al. 2023 [18]	Prospective	Denmark	ED	75	41 (55)	65	28	1.5	1.4	Board certified physician	CTPA or V/Q scan
Lieveld et al. 2022 [19]	Prospective	Turkey	ICU	70	14 (20)	67.5	32.8	NA	2.21	Experienced ultrasound physician	CTPA
Nazerian et al. 2014 [11]	Prospective	Italy	ED	357	188 (52.7)	71 ± 14.4	30.8	NA	NA	Emergency staff physician and residents physicians	CTPA

CTPA: computed tomography pulmonary angiography; ED: emergency department; ICU: intensive care unit; V/Q: ventilation perfusion scan

CTPA was the primary reference standard used. One study utilized both CTPA and V/Q scan [18]. The main alternative diagnoses observed in addition to PE were, pneumonia, heart failure, COPD, acute coronary syndrome and muscular chest pain. Additionally, one study focused exclusively on patients with COVID-19 [19].

Distinct findings were observed across the included studies for the diagnosis of PE using ultrasound. With respect to lung ultrasound, three studies [11, 18, 19] identified PE by noting subpleural wedge-shaped, triangular, or rounded hypoechoic lesions. One study [19] used two or more subpleural consolidations ≥ 1 cm as the diagnostic criteria for PE. The cardiac ultrasound parameters included in all studies were right ventricle (RV) dilatation, D-shaped interventricular septum and visualization of a thrombus in the right cardiac chambers. Venous ultrasound consistently showed the absence of vein collapse during compression with or without a visible intravascular thrombus, which is indicative of deep vein thrombosis (DVT). A comprehensive list of ultrasound features for PE diagnosis is provided in Table 2.

**Table 2:** Ultrasound features for pulmonary embolism diagnosis

Study	Lung ultrasound	Cardiac ultrasound	Vascular ultrasound
Aktürk 2017 [17]	Subpleural wedge-shaped, triangular, or rounded hypoechoic lesions	Right ventricular dilatation, high pulmonary arterial pressure, and D-shaped interventricular septum (D-sign) Thrombi in the right cavity	Lack of vein collapse during compression Visible intravascular thrombi
Falster et al. 2023 [18]	Subpleural wedge-shaped, triangular, or rounded hypoechoic lesions	60/60-sign (pulmonary valve acceleration time < 60 ms with tricuspid regurgitation peak gradient < 60 mmHg) D-sign McConnell’s sign (akinesia of the RV free wall with preserved apical motion) TAPSE	Lack of vein collapse during compression Visible intravascular thrombi
Lieveld et al. 2022 [19]	Two or more subpleural consolidations (≥ 1 cm)	Right ventricular strain (RVS) observed as flattening or bowing of the interventricular septum (D-sign) Assessments included RV to LV basal end diastolic diameter ratio (≥ 1) Presence of thrombi in the right ventricular cavity	Lack of vein collapse during compression Visible intravascular thrombi
Nazerian et al. 2014 [11]	Subpleural wedge-shaped, triangular, or rounded hypoechoic lesions	Right ventricular dilatation indicated by abnormal RV/LV ratio or RV end-diastolic diameter Presence of thrombi in the right ventricular cavity	Lack of vein collapse during compression Visible intravascular thrombi

*LV* left ventricle, *RV* right ventricle, *TAPSE* tricuspid annular plane systolic excursion

### Quality assessment

**Fig. 2:**
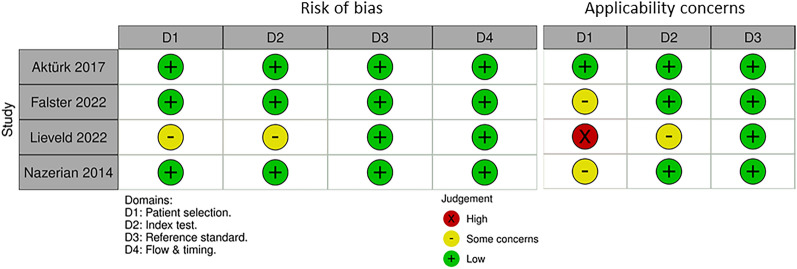
Quality assessment of included studies. Overall, the assessment indicated a generally low risk of bias, with one study demonstrating a high risk of bias

Figure 2 illustrates the quality assessment of all included studies evaluated using the QUADAS-2 tool. Overall, the assessment indicated a generally low risk of bias, with one study [19] demonstrating a high risk of bias due to concerns in two domains and high applicability concerns in domain 1. A full description of all four domains for each study is provided in Supplementary Method 2.

**Fig. 3:**
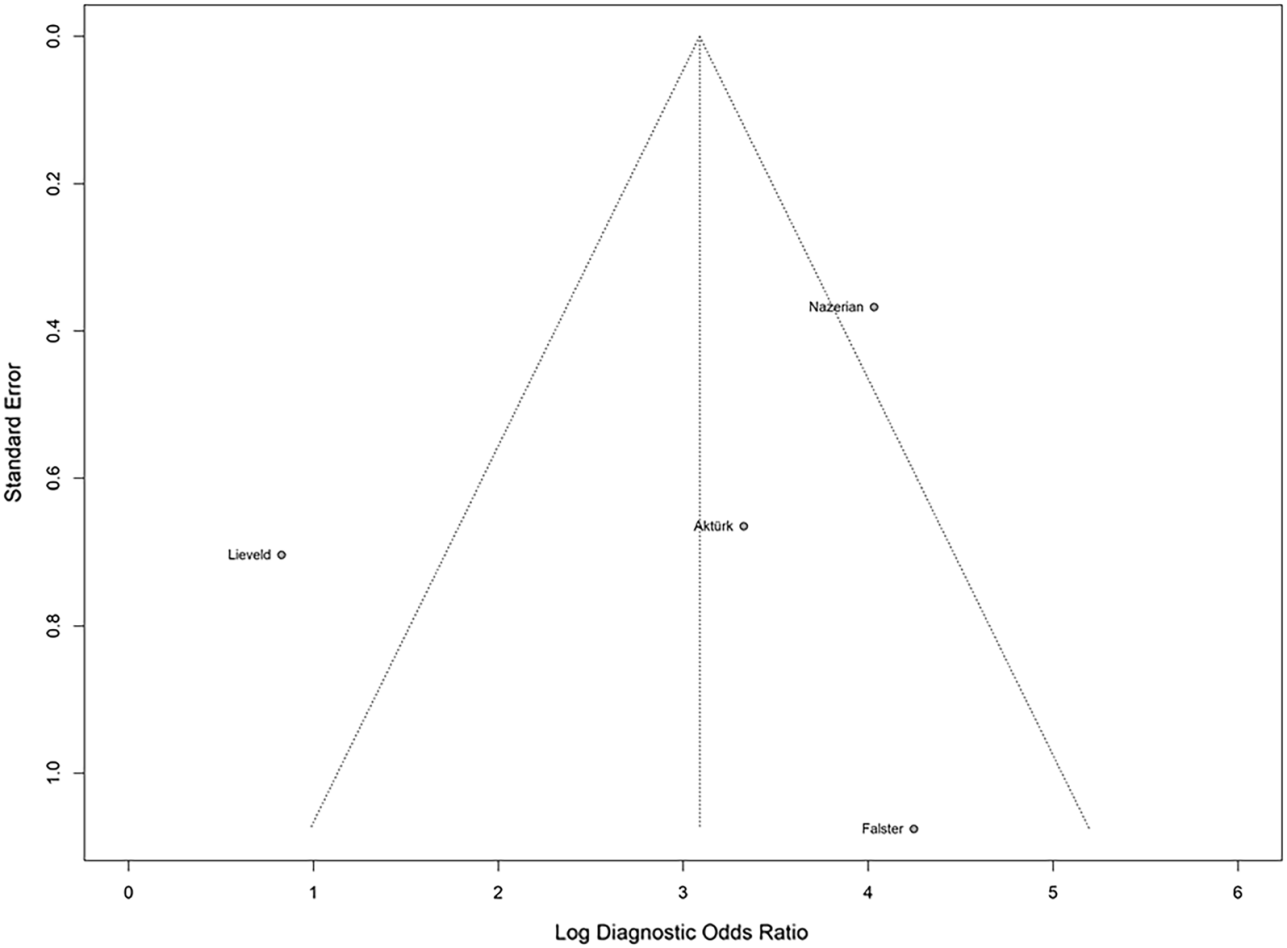
Funnel plot asymmetry test for publication bias using Deek’s model revealed slight asymmetry

The funnel plot (Fig. 3) revealed slight asymmetry upon visual inspection.However, Egger’s regression test could not be conducted because of the limited number of studies included.

### Diagnostic accuracy of multiorgan POCUS

**Fig. 4:**
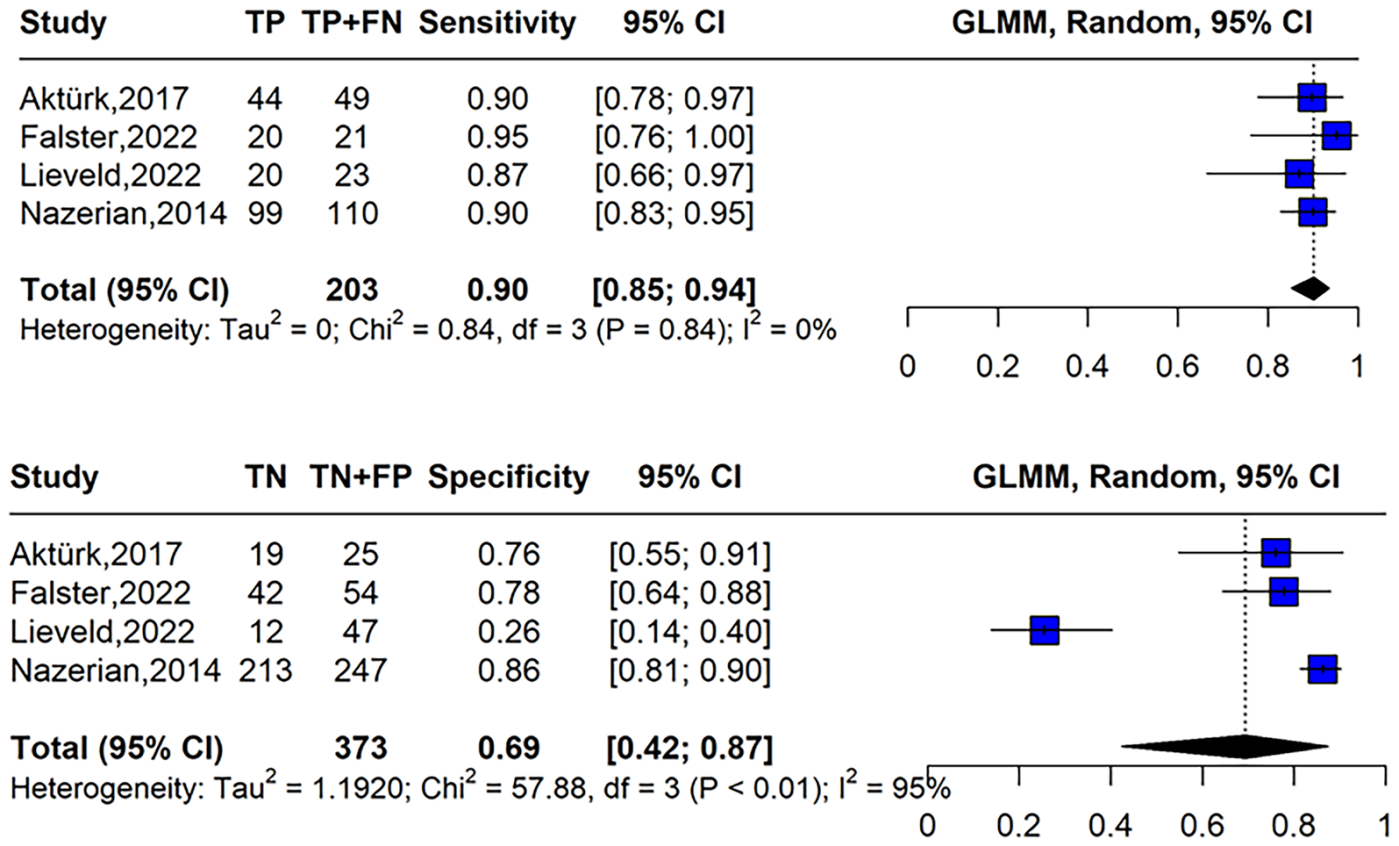
Forest plot of pooled sensitivity (top) and specificity (bottom) of multi-organ point-of-care ultrasound for pulmonary embolism diagnosis

**Fig. 5:**
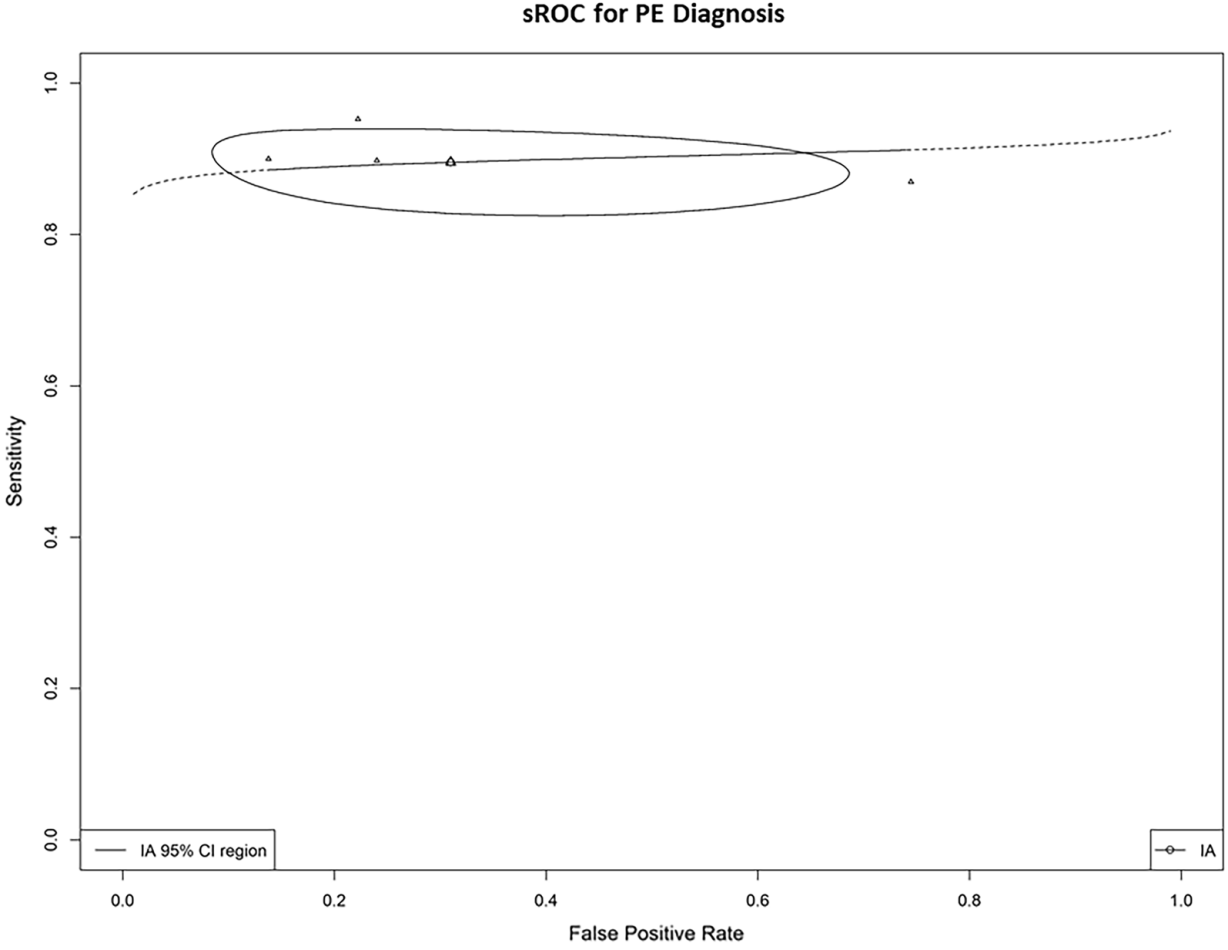
Summary receiver operator curve of diagnostic performance of multi-organ point-of-care ultrasound for pulmonary embolism diagnosis. PE: pulmonary embolism

Figure 4 presents the summary estimates of sensitivity and specificity for each individual study. These estimates provide insight into the diagnostic performance of multi-organ ultrasound in detecting PE across the included studies. The pooled DOR was 25.3 (95% CI 4.43–82.9). The pooled sensitivity and specificity of the four studies for the diagnosis of PE were 0.90 (95% CI 0.85–0.94; I^2^ = 0%) and 0.69 (95% CI 0.42–0.87; I^2^ = 95%), respectively, with a PLR of 3.35 (95% CI 1.43–8.02) and a NLR of 0.16 (95% CI 0.08–0.32).The SROC curve had an AUC of 0.89 (Fig. 5, 95% CI 0.81–0.94). When omitting the study with high risk of bias, the pooled sensitivity was 0.91 (95% CI 0.85–0.94; I^2^ = 0%), specificity 0.84 (95% CI 0.73–0.90; I^2^ = 44%), PLR of 5.17 (95% CI 3.5–7.55) and NLR of 0.118 (95% CI 0.07–0.18).

### Sensitivity analyses

In the leave-one-out sensitivity analyses, the overall pooled sensitivity remained stable across different iterations of the meta-analysis with individual studies omitted (Supplementary Fig. 1). The overall specificity also showed consistent values across interactions, however,there was a significant decrease in heterogeneity (I^2^ = 44%) when one specific study was excluded [18].

## Discussion

In this meta-analysis encompassing 594 patients from 4 studies, we evaluated the diagnostic accuracy of multi-organ POCUS for PE in critical care setting. The results demonstrated a DOR of 25.3 with a pooled sensitivity and specificity of 90% and 69%, respectively. Additionally, the SROC curve revealed an AUC of 0.89, indicating high test accuracy. To our knowledge, this is the first meta-analysis assessing the accuracy of a multi-organ POCUS approach—combining lung, cardiac and venous ultrasound- for the diagnosis of PE in critically ill patients.

PE is a common and potentially fatal condition if left untreated [1, 20]. Consequently, the diagnostic approach must be both efficient and judicious, balancing the need to avoid unnecessary testing while ensuring timely diagnosis and treatment to reduce morbidity and mortality.

The latest European Society of Cardiology guidelines [21] suggest that the optimal diagnostic strategy to confirm or exclude PE involves a combination of pretest probability assessment using validate tools such Wells score or the Geneva score, plasma D-dimer measurement and CTPA. While CTPA is currently regarded as the gold-standard diagnostic method, it has several limitations, including high cost, lack of 24-h availability in many hospitals, particularly in limited-resource settings, and the need for patient transportation to the radiology department.This latter requirement poses a significant risk in hemodynamically unstable patients. These factors, in addition to presenting risks inherent to the method, such as radiation exposure and potential allergic reactions to contrast agents, can make PE diagnosis challenging in the critically ill patients. Given these challenges, POCUS has emerged as a valuable diagnostic tool. It is non-invasive, widely available and can be performed at the bedside by the treating physician. Lung ultrasound, which can detect sonographic signs of pulmonary infarction, has been validated and is recommended by experts as a promising alternative when CTPA is not feasible [22]. The use of lung ultrasonography in emergency (BLUE) protocol was the first dual-organ ultrasound approach, combining lung and venous ultrasound, and demonstrated a sensitivity of 81% and a specificity of 99% for PE diagnosis [23].

Despite its advantages, ultrasound assessments typically have limited utility in conclusively ruling out PE due to their relatively low sensitivity, even under optimal insonation conditions. To address this limitation, Nazerian et al. [11] introduced a multi-organ POCUS approach that integrates cardiac, lung and venous ultrasound with the Wells score and D-dimer testing. This approach demonstrated promising potential for ruling out PE and may serve as a valid alternative when CTPA is unavaliable or contraindicated.

Previous meta-analyses evaluating the accuracy of ultrasound for PE diagnosis have primarily focused on single-organ insonation or dual-organ protocols [10, 24–26]. The potential to enhance the accuracy of a multi-organ POCUS approach lies in its ability to improve sensitivity—combining negative findings from lung, cardiac, and venous ultrasounds yields a higher negative predictive value than any single-organ ultrasound alone. POCUS may be charged by the crucial role of becoming the valid alternative to CTPA. Furthermore, incorporating likelihood ratios into pretest probability assessments and integrating multi-organ POCUS with clinical prediction tools, such as the Wells score, has been shown to enhance diagnostic efficiency. This approach has the potential to significantly reduce the number of unnecessary CTPA scans in emergency settings [27]. Another study published by Falster and colleagues [28] demonstrated a substantial reduction in referral do diagnostic imaging in suspected PE when a multi-organ POCUS approach was employed. Therefore, extending the ultrasound assessment to multiple organs may further improve the accuracy of pretest probability calculations, making the multi-organ POCUS approach a valuable and potentially a cost-effective diagnostic strategy.

This review has several strengths. To our knowledge, this is the first meta-analysis to evaluate the accuracy of a combined lung, cardiac and venous POCUS approach in ruling-in and ruling-out PE, addressing a gap left by previous reviews that focused on single or dual-organ evaluations. Additionally, we implemented a rigorous and comprehensive search strategy across multiple databases, complemented by backward and forward citation tracking. Only prospective studies were included, most of which demonstrated a low risk of bias.

However, our study has also several limitations. First, despite a thorough search and selection process, only four studies met the inclusion criteria. This small sample size may limit the generalizability of the findings and reduce the statistical power to detect smaller effects. Second, significant heterogeneity in specificity could affect the applicability of the results. Notably, the leave-one-out sensitivity analysis revealed a marked decrease in heterogeneity in the specificity plot when the study by Lieveld et al. was excluded. This particular study focused exclusively in COVID-19 population, which has a greater risk for both PE and other lung [29] and cardiac ultrasound [30] findings that could mimic PE, leading to elevated false-positive rates. Moreover, the lung ultrasound criteria for diagnosing PE in this study were not specific (Table 2) as evidenced by its notably low specificity of 25%, which contrasts sharply with the findings of other studies. Third, it is important to consider the characteristics of the study populations included in our meta-analysis. Notably, three out of the four studies included were conducted in emergency department (ED) settings. This predominance of ED-based studies could introduce bias, potentially skewing the diagnostic performance of POCUS in favor of more favorable PLRs and NLRs. As a result, the accuracy reported in our meta-analysis may be optimized for acute, high-suspicion clinical scenarios rather than for routine or lower-suspicion settings. Finally, variability in ultrasound equipment and operator expertise across the included studies could also influence diagnostic accuracy. These factors should be considered when interpreting the results and considering their applicability to broader clinical contexts.

## Conclusion

In our study, multi-organ POCUS has demonstrated high accuracy for ruling in or out PE in critically ill patients, offering a valuable, cost-effective alternative to traditional imaging modalities, especially in resource-limited environments. Further research is needed to validated these findings across different patient populations.

## Supplementary Information

Additional file 1.

## Data Availability

No datasets were generated or analysed during the current study.

## Abbreviation lists

AUC: Area under the curve
CI: Confidence interval
CTPA: Computed tomographic pulmonary angiography
DOR: Diagnostic odds ratio
DVT: Deep vein thrombosis
I^2^: Inconsistency index
ITT: Intention-to-treat
NLR: Negative likelihood ratio
PE: Pulmonary embolism
PLR: Positive likelihood ratio
POCUS: Point-of-care ultrasound
PRISMA: Preferred reporting items for systematic reviews and meta-analysis
QUADAS-2: Quality assessment of diagnostic accuracy studies-2
RV: Right ventricle
SROC: Summary receiver operating characteristic
V/Q: Ventilation/perfusion
