# Supplementary material for: Diagnostic accuracy of multi-organ point-of-care ultrasound for pulmonary embolism in critically ill patients: a systematic review and meta-analysis
Source: Crit Care. 2025 Apr 23;29:162. doi: 10.1186/s13054-025-05359-x (PMC12020239; doi:10.1186/s13054-025-05359-x)
Supplement: Supplementary file 1 — Additional file 1. [file 13054_2025_5359_MOESM1_ESM.docx]

**SUPPLEMENTARY MATERIAL**

**Table of Contents**

**Supplemental Methods 1 – Detailed Search Strategy3**

**Supplemental Methods 2 – Risk of Bias - QUADAS-24**

**Supplementary Figure 1 10**

**Supplementary Table 1.........................................................................................................11**

**PRISMA Checklist 12**

**Supplemental Methods 1 – Detailed Search Strategy**

| **Pubmed** | (''pulmonary embolism'' OR "pulmonary embolism"[MeSH Terms] OR ''pulmonary thromboembolism'' OR PE) AND ("point-of-care systems"[MeSH Terms] OR ''ultrasound'' OR ''ultrasonography'' OR "ultrasonography"[MeSH Terms] OR ''point-of-care ultrasound'' OR POCUS) AND ("computed tomography pulmonary angiography" OR ''pulmonary angiography'' OR CTPA OR ''computed tomography pulmonary angiogram'' OR CTA) AND (accuracy OR ''diagnostic accuracy'' OR sensitivity OR specificity OR "sensitivity and specificity"[MeSH Terms]) |
| --- | --- |
| **Embase** | ('pulmonary embolism'/exp OR 'pulmonary thromboembolism' OR PE) AND ('point of care testing'/exp OR ultrasound OR ultrasonography OR 'bedside ultrasound' OR POCUS) AND ('computed tomography pulmonary angiography'/exp OR 'pulmonary angiography' OR CTPA OR 'computed tomography pulmonary angiogram' OR CTA) AND (accuracy OR 'diagnostic accuracy' OR sensitivity OR specificity) |
| **Cochrane Library** | ("pulmonary embolism" OR "pulmonary thromboembolism" OR PE) AND ("point of care systems" OR ultrasound OR ultrasonography OR "point of care ultrasound" OR POCUS) AND ("computed tomography pulmonary angiography" OR "pulmonary angiography" OR CTPA OR "computed tomography pulmonary angiogram" OR CTA) AND (accuracy OR "diagnostic accuracy" OR sensitivity OR specificity) |
| **Scopus** | (TITLE-ABS-KEY("pulmonary embolism" OR "pulmonary thromboembolism" OR PE) AND TITLE-ABS-KEY("point-of-care systems" OR "ultrasound" OR "ultrasonography" OR "point-of-care ultrasound" OR POCUS) AND TITLE-ABS-KEY("computed tomography pulmonary angiography" OR "pulmonary angiography" OR CTPA OR "computed tomography pulmonary angiogram" OR CTA) AND TITLE-ABS-KEY(accuracy OR "diagnostic accuracy" OR sensitivity OR specificity)) |

**Supplemental Methods 2 – Risk of Bias - QUADAS-2**

| **Akturk 2017** |
| --- |
| 1. ****Patient Selection****  - **Risk of Bias:** Low to moderate. The study includes consecutive patients, which reduces selection bias. However, only patients with moderate to high clinical suspicion of PE were included, which might not represent the wider population of patients suspected of having PE. The inclusion and exclusion criteria are clearly defined, which is a positive aspect - **Applicability Concerns:** Low. The study specifically targets patients with a moderate to high clinical suspicion of pulmonary embolism (PE), which aligns well with the typical clinical setting where such diagnostic methods would be used. |
| 2. ****Index Test****   - **Risk of Bias:** Low. The ultrasonography was performed by an experienced chest physician, and it seems that the test was conducted without knowledge of the reference standard outcomes (multislice CTPA results). - **Applicability Concerns:** Low. Multi-organ ultrasonography is evaluated as a potentially useful diagnostic tool for PE in emergency settings, where rapid and non-invasive diagnostics are crucial. The use of this diagnostic approach fits well with clinical needs in emergency departments and potentially in other acute care settings where CTPA may be unavailable or delayed. |
| 3. ****Reference Standard****  - **Risk of Bias:** Likely low. Multislice CTPA is considered a highly reliable method for diagnosing PE and is used as the reference standard. The interpretation of CTPA was performed by a radiologist blinded to the results of the ultrasound and other examinations, reducing the risk of diagnostic review bias. - **Applicability Concerns:** Low. Multislice CTPA is widely accepted and used as the reference standard for diagnosing PE. The use of CTPA in this study is highly applicable to real-world clinical settings where PE is suspected, reinforcing the external validity of the study findings in similar healthcare contexts. |
| 4. ****Flow and Timing****  - **Risk of Bias:** Low. All patients underwent the index test and reference standard (CTPA) within 24 hours of each other, which is optimal to reduce the possibility of disease progression affecting the test outcomes. |
| Conclusion: The study's design and methodology are well-aligned with clinical practices in emergency settings, particularly for patients who are suspected of having non-massive pulmonary embolism based on moderate to high clinical suspicion. The primary area of applicability concern lies in the patient selection, as the results may not extend as reliably to populations with low clinical suspicion of PE or different healthcare settings such as outpatient clinics or primary care, where the prevalence and presentation of PE may differ. Thus, while the study's findings are robust for its intended clinical context, caution should be exercised when extrapolating these results to broader or different patient populations. |
| **Falster 2022** |
| **1. Patient Selection**   - **Risk of Bias:** Low. The study includes patients with a clinical suspicion of PE referred for further diagnostic testing, which aligns well with the aim of assessing a diagnostic tool in a relevant clinical context. The use of a convenience sample is typical for diagnostic accuracy studies but does limit random selection. - **Applicability Concerns:** Moderate. While the patient selection criteria are appropriate for the study’s objectives, the use of a convenience sample and exclusion of patients with known contrast allergy or renal failure may not reflect the full spectrum of patients typically evaluated for PE in general practice. This could affect the generalizability of the study's findings to all suspected PE cases. |
| **2. Index Test**   - **Risk of Bias:** Low. The ultrasound examinations were performed blinded to the results of the reference standard, which minimizes interpretation bias. The standardized protocol and the expertise of the operator enhance the reliability of the ultrasound findings. - **Applicability Concerns:** Low. The bespoke multiorgan ultrasound approach is highly relevant and applicable in clinical settings, especially in emergency and acute care where quick, non-invasive diagnostic tools are valuable. The study supports the use of this approach in similar healthcare environments. |
| **3. Reference Standard**   - **Risk of Bias:** Low. Both CTPA and ventilation/perfusion scintigraphy are well-established reference standards for diagnosing PE, and the blinded assessment by radiologists and nuclear medicine physicians ensures an unbiased evaluation of these tests. - **Applicability Concerns:** Low. These reference standards are the gold standard tests for PE diagnosis in clinical practice, ensuring that the study's findings are applicable to clinical settings where such diagnostic capabilities are available. |
| **4. Flow and Timing**   - **Risk of Bias:** Low. All patients underwent the index test and reference standard within a closely controlled time frame, ensuring that changes in clinical status did not affect the diagnostic accuracy. |
| **Conclusion:** The study by Falster et al. demonstrates a low risk of bias and generally low concerns about the applicability of its findings, indicating that the results are both robust and relevant for clinical use. The bespoke multiorgan ultrasound approach evaluated in this study is particularly suited for emergency and acute care settings, where rapid, accurate diagnosis of PE is crucial. However, the potential limitations due to the convenience sampling and exclusion criteria should be considered when generalizing these results to broader populations or different clinical contexts. |
| **Lieveld 2022** |
| **1. Patient Selection**   - **Risk of Bias:** Moderate. The study's focus on a specific subgroup of critically ill COVID-19 patients in an ICU setting may introduce a selection bias. While the consecutive sampling of patients minimizes some risk, the unique characteristics of this population (e.g., higher baseline risk for thrombotic events, altered pulmonary imaging due to COVID-19-related changes) could affect the generalizability of the results and the interpretation of how POCUS performs in a broader group of patients suspected of PE. - **Applicability Concerns:** High.The results are highly specific to critically ill COVID-19 patients, who are known to have a higher incidence of both pulmonary embolism and other pulmonary complications that might mimic or obscure PE findings on imaging studies. This specificity limits the applicability to the general population or to patients outside of similar critical care settings. Additionally, the underlying pathophysiological changes in COVID-19 that impact lung imaging may not be present in other patient groups, affecting the diagnostic utility of POCUS in non-COVID-19 contexts. |
| **2. Index Test**   - **Risk of Bias:** Uncertain. The POCUS was performed by experienced operators and was carried out blind to the CTPA results, minimizing interpretation bias. The procedures for POCUS were standardized across patients. However, the expertise level of operators (experienced ultrasound physicians) are not clear, neither the training procedure. - **Applicability Concerns:** Uncertain. While the study supports POCUS as a feasible initial diagnostic approach, the expertise level of operators (experienced ultrasound physicians) are not clear, neither the training procedure, which might limit the generalizability to settings where such expertise is not readily available. |
| **3. Reference Standard**   - **Risk of Bias:** Low. CTPA is the accepted reference standard for diagnosing pulmonary embolism. Radiologists had access to clinical information but not to the POCUS results, reducing potential bias. - **Applicability Concerns:** Low. Since CTPA is the standard reference in diagnosing PE, the results are highly applicable to clinical practice where CTPA is available and feasible to perform. |
| **4. Flow and Timing**   - **Risk of Bias:** Low. The POCUS and reference standard tests were performed within 24 hours of each other, minimizing the time for clinical change that could affect the diagnostic accuracy. |
| **Conclusion:** The applicability of the study findings is more limited when considering use outside of similar critical care settings or among non-COVID-19 populations. This context should be carefully considered when integrating the study's conclusions into broader diagnostic protocols. The utility of multi-organ POCUS may still be significant in similar high-risk groups but may not directly translate to broader patient populations or settings without adaptation or further validation. |
| **Nazerian 2014** |
| **1. Patient Selection**   - **Risk of Bias:** Low to moderate. Patients were selected based on having a Wells score greater than 4 or a positive D-dimer, which introduces some risk of bias as it excludes a large portion of patients with suspected PE, potentially leading to spectrum bias. However, this is what is recommended in the real-world clinical practice. Only moderate-high risk patients for PE (as evaluated by wells score) are evaluated with imaging diagnostics exams. - **Applicability:** Low to moderate The study’s applicability is limited to patients with high pretest probability of PE or those with significant D-dimer elevation. This selection criterion may not be representative of all patients with suspected PE, such as those with lower clinical suspicion or those in different settings like primary care or less acute hospital settings. However, Only moderate-high risk patients for PE (as evaluated by wells score) are evaluated with imaging diagnostics exams in the real-world clinical practice. |
| **2. Index Test**   - **Risk of Bias:** Low. The ultrasonography was performed by a variety of trained sonographers and blinded to the MCTPA results, minimizing interpretation bias. The standardized and systematic approach to ultrasonography across multiple organs reduces the likelihood of operator-dependent variability. - **Applicability Concerns:** Low. The use of multiorgan ultrasonography is highly relevant for emergency departments where quick, non-invasive diagnostic alternatives to CT are needed. Its effectiveness in this setting supports its applicability in similar clinical environments. |
| **3. Reference Standard**   - **Risk of Bias:** Low. MCTPA is the gold standard for diagnosing PE, and its use as the reference standard is appropriate. The radiologists were blinded to the ultrasonography results, which reduces diagnostic review bias. - **Applicability Concerns:** Low. MCTPA is widely recognized and used in clinical practice for the diagnosis of PE, making the study’s findings applicable to settings where MCTPA is available. |
| **4. Flow and Timing**   - **Risk of Bias:** Low. The ultrasonography and MCTPA were performed in a timely manner, with all ultrasound examinations done before MCTPA and typically within a tight window, which minimizes the risk of disease progression affecting the test outcomes. |
| **Conclusion:** The study by Nazerian et al. presents a generally low risk of bias in the index test, reference standard, and flow and timing domains but shows low to moderate risk in patient selection. This might affect the generalizability of the findings to the entire population of patients with suspected PE, especially those with lower clinical suspicion or those seen in different medical settings. The focus on higher-risk patients somewhat limits the broader applicability of the results, although within the emergency department context, the findings remain highly relevant and useful. |

**Supplementary Figure 1 – Leave-one-out sensitive analyses for sensitivity and specificity**


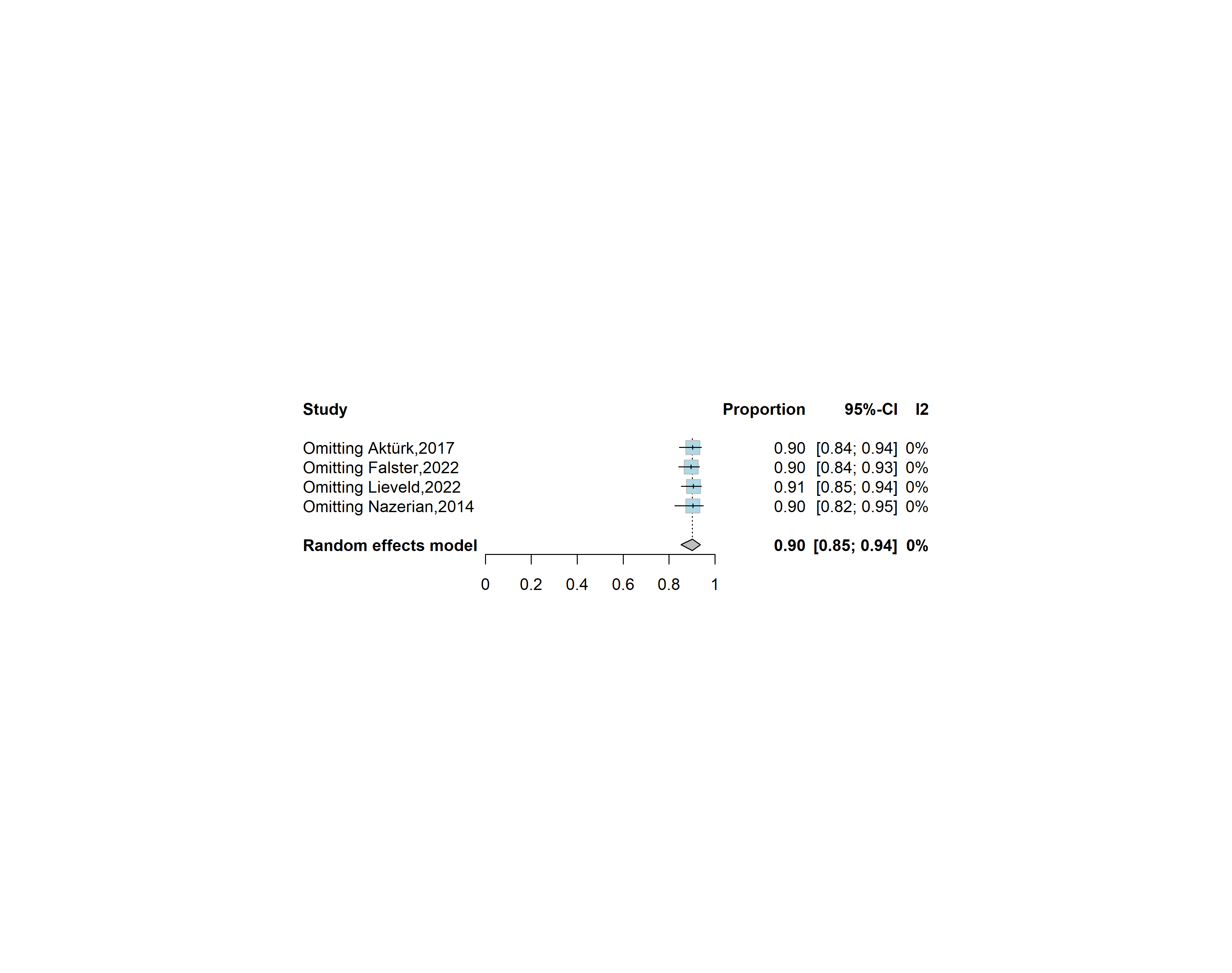


**Sensitivity**

**Specificity**


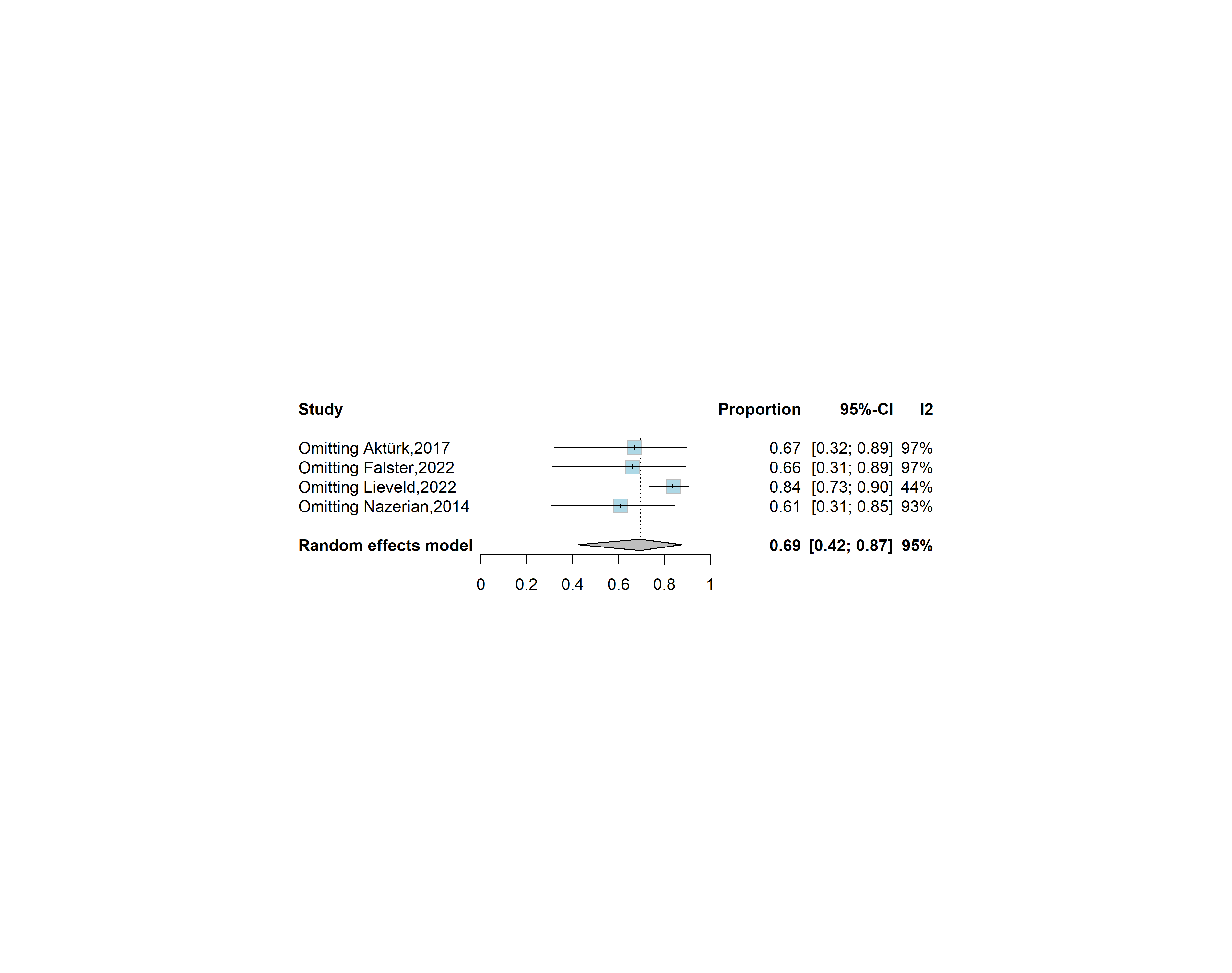


**SUPPLEMENTARY TABLE 1**

**Lieveld 2022**

**Falster 2023**

**Nazerian 2014**

**Akturk 2017**

| **POCUS** | **Sensitivity** | **Specificity** | **PLR** | **NLR** |
| --- | --- | --- | --- | --- |
| **Lung US** | 65.3% | 92% | **8.16** | **0.38** |
| **Cardiac US** | 12.3% | 100% | **NA** | **0.88** |
| **Vein US** | 51% | 88% | **4.25** | **0.56** |
| **Lung US** | 61.9% | 94.4% | **11** | **0.4** |
| **Cardiac US** | 38.1% | 94.4% | **6.8** | **0.6** |
| **Vein US** | 28.6% | 100% | **NA** | **0.71** |
| **Lung US** | 70.8% | 36.3% | **1.1** | **0.8** |
| **Cardiac US** | 40% | 83% | **2.35** | **0.72** |
| **Vein US** | 24% | 88.8% | **2.14** | **0.86** |
| **Lung US** | 60.9% | 95.9% | **15** | **0.4** |
| **Cardiac US** | 32.7% | 90.9% | **3.6** | **0.7** |
| **Vein US** | 52.7% | 97.6% | **21.7** | **0.5** |

| **Section and Topic** | **Item #** | **Checklist item** | **Location where item is reported** |
| --- | --- | --- | --- |
| **TITLE** | | |  |
| Title | 1 | Identify the report as a systematic review. | Pg 1 |
| **ABSTRACT** | | |  |
| Abstract | 2 | See the PRISMA 2020 for Abstracts checklist. |  |
| ***INTRODUCTION*** | | |  |
| Rationale | 3 | Describe the rationale for the review in the context of existing knowledge. | Pg 1 |
| Objectives | 4 | Provide an explicit statement of the objective(s) or question(s) the review addresses. | Pg 1 |
| **METHODS** | | |  |
| Eligibility criteria | 5 | Specify the inclusion and exclusion criteria for the review and how studies were grouped for the syntheses. | Pg 2 |
| Information sources | 6 | Specify all databases, registers, websites, organisations, reference lists and other sources searched or consulted to identify studies. Specify the date when each source was last searched or consulted. | Pg 2 |
| Search strategy | 7 | Present the full search strategies for all databases, registers and websites, including any filters and limits used. | Pg 3 (Supplement) |
| Selection process | 8 | Specify the methods used to decide whether a study met the inclusion criteria of the review, including how many reviewers screened each record and each report retrieved, whether they worked independently, and if applicable, details of automation tools used in the process. | Pg 3 |
| Data collection process | 9 | Specify the methods used to collect data from reports, including how many reviewers collected data from each report, whether they worked independently, any processes for obtaining or confirming data from study investigators, and if applicable, details of automation tools used in the process. | Pg 3 |
| Data items | 10a | List and define all outcomes for which data were sought. Specify whether all results that were compatible with each outcome domain in each study were sought (e.g. for all measures, time points, analyses), and if not, the methods used to decide which results to collect. | Pg 3 |
|  | 10b | List and define all other variables for which data were sought (e.g. participant and intervention characteristics, funding sources). Describe any assumptions made about any missing or unclear information. | Pg 3 |
| Study risk of bias assessment | 11 | Specify the methods used to assess risk of bias in the included studies, including details of the tool(s) used, how many reviewers assessed each study and whether they worked independently, and if applicable, details of automation tools used in the process. | Pg 3 |
| Effect measures | 12 | Specify for each outcome the effect measure(s) (e.g. risk ratio, mean difference) used in the synthesis or presentation of results. | Pg 3 |
| Synthesis methods | 13a | Describe the processes used to decide which studies were eligible for each synthesis (e.g. tabulating the study intervention characteristics and comparing against the planned groups for each synthesis (item #5)). | Pg 3,4 |
|  | 13b | Describe any methods required to prepare the data for presentation or synthesis, such as handling of missing summary statistics, or data conversions. | Pg 3,4 |
|  | 13c | Describe any methods used to tabulate or visually display results of individual studies and syntheses. | Pg 3,4 |
|  | 13d | Describe any methods used to synthesize results and provide a rationale for the choice(s). If meta-analysis was performed, describe the model(s), method(s) to identify the presence and extent of statistical heterogeneity, and software package(s) used. | Pg 2-4 |
|  | 13e | Describe any methods used to explore possible causes of heterogeneity among study results (e.g. subgroup analysis, meta-regression). | Pg 3-5/ Pg 10 (supplement) |
|  | 13f | Describe any sensitivity analyses conducted to assess robustness of the synthesized results. | Pg 5 / Pg 10 (supplement) |
| Reporting bias assessment | 14 | Describe any methods used to assess risk of bias due to missing results in a synthesis (arising from reporting biases). | Pg 5, figure 3 |
| Certainty assessment | 15 | Describe any methods used to assess certainty (or confidence) in the body of evidence for an outcome. | Pg 4-6 |
| **RESULTS** | | |  |
| Study selection | 16a | Describe the results of the search and selection process, from the number of records identified in the search to the number of studies included in the review, ideally using a flow diagram. | Pg 4 |
|  | 16b | Cite studies that might appear to meet the inclusion criteria, but which were excluded, and explain why they were excluded. | Figure 1 |
| Study characteristics | 17 | Cite each included study and present its characteristics. | Table 1 |
| Risk of bias in studies | 18 | Present assessments of risk of bias for each included study. | Pg 4 (supplement)  Figure 2 |
| Results of individual studies | 19 | For all outcomes, present, for each study: (a) summary statistics for each group (where appropriate) and (b) an effect estimate and its precision (e.g. confidence/credible interval), ideally using structured tables or plots. | Pg,5, Figure 4 |
| Results of syntheses | 20a | For each synthesis, briefly summarise the characteristics and risk of bias among contributing studies. | Pg 4-10 (supplement) |
|  | 20b | Present results of all statistical syntheses conducted. If meta-analysis was done, present for each the summary estimate and its precision (e.g. confidence/credible interval) and measures of statistical heterogeneity. If comparing groups, describe the direction of the effect. | Pg,5, Figure 4 and 5 |
|  | 20c | Present results of all investigations of possible causes of heterogeneity among study results. | Pg 5 |
|  | 20d | Present results of all sensitivity analyses conducted to assess the robustness of the synthesized results. | Pg 10 (supplement) |
| Reporting biases | 21 | Present assessments of risk of bias due to missing results (arising from reporting biases) for each synthesis assessed. | Pg 4-5, Figure 3 |
| Certainty of evidence | 22 | Present assessments of certainty (or confidence) in the body of evidence for each outcome assessed. | Pg 5 |
| **DISCUSSION** | | |  |
| Discussion | 23a | Provide a general interpretation of the results in the context of other evidence. | Pg 6 |
|  | 23b | Discuss any limitations of the evidence included in the review. | Pg 7,8 |
|  | 23c | Discuss any limitations of the review processes used. | Pg 7,8 |
|  | 23d | Discuss implications of the results for practice, policy, and future research. | Pg 7,8 |
| **OTHER INFORMATION** | | |  |
| Registration and protocol | 24a | Provide registration information for the review, including register name and registration number, or state that the review was not registered. | Pg 2 |
|  | 24b | Indicate where the review protocol can be accessed, or state that a protocol was not prepared. | Pg 2 (PROSPERO) |
|  | 24c | Describe and explain any amendments to information provided at registration or in the protocol. | Pg 2 |
| Support | 25 | Describe sources of financial or non-financial support for the review, and the role of the funders or sponsors in the review. | Title page |
| Competing interests | 26 | Declare any competing interests of review authors. | Title page |
| Availability of data, code and other materials | 27 | Report which of the following are publicly available and where they can be found: template data collection forms; data extracted from included studies; data used for all analyses; analytic code; any other materials used in the review. | Supplementary material |
